# Elucidating the genetic relationship between ulcerative colitis and diabetic kidney disease: a bidirectional Mendelian randomization study

**DOI:** 10.3389/fendo.2024.1435812

**Published:** 2024-08-15

**Authors:** Yaping Guo, Hangxing Yu, Ying Li, Taijun Zhang, Weijian Xiong, Xili Wu

**Affiliations:** ^1^ Xi’an Jiaotong University, Xi’an, China; ^2^ Yulin Hospital of Traditional Chinese Medicine, Yulin, China; ^3^ Chongqing Hospital of Traditional Chinese Medicine, Chongqing, China

**Keywords:** ulcerative colitis, diabetic kidney disease, Mendelian randomization, genetic causality, genome-wide association studies (GWAS)

## Abstract

**Introduction:**

Ulcerative colitis (UC) and diabetic kidney disease (DKD) are chronic disorders with multifaceted pathogenesis, posing significant challenges in clinical management. While substantial efforts have been made to investigate the individual causes of these diseases, the interplay between UC and DKD is not well understood. This study aims to elucidate the genetic association between UC and DKD through Mendelian randomization (MR) analysis, offering new insights into common biological pathways and potential clinical implications.

**Methods:**

We conducted a bidirectional two-sample MR study utilizing data from large-scale genome-wide association studies (GWAS) for both UC and DKD. Instrumental variables (IVs) were meticulously selected according to genome-wide significance and stringent statistical criteria, ensuring robust causal inference. Various MR methodologies, including inverse variance weighting (IVW), were employed to assess the causal relationships between UC and DKD. Sensitivity analyses were also performed to validate the robustness of our findings.

**Results:**

Our analysis revealed a significant causal relationship between genetic predisposition to UC and increased susceptibility to DKD. Specifically, individuals with a genetic susceptibility to UC exhibited a 17.3% higher risk of developing DKD. However, we found no evidence of a causal link between DKD and the risk of developing UC. Additionally, we identified shared genetic risk factors and molecular pathways linking UC and DKD, thereby highlighting potential therapeutic targets.

**Discussion:**

This study underscores the intricate genetic interplay between UC and DKD, suggesting that individuals with UC may be at an elevated risk for developing DKD. Understanding these shared genetic pathways could facilitate the development of early detection strategies and targeted interventions for individuals at risk of DKD. Ultimately, these insights could lead to improved clinical outcomes for patients suffering from both conditions.

## 1 Introduction

Ulcerative colitis (UC), a major form of inflammatory bowel disease (IBD), is a chronic inflammatory condition of the colonic mucosa affecting the rectum and colon to varying degrees. Its pathogenesis is closely linked to genetic susceptibility, microbiota, and immune dysregulation (1). Renal involvement is now common in patients with IBD, including UC (2). A cohort study including renal biopsies from IBD patients showed that out of 896 IBD patients, 218 (24.3%) had renal involvement, of which 161 (73.85%) were UC patients (3). Several national studies have shown that IBD patients have a higher risk of developing type 2 diabetes than the general population, not solely attributable to corticosteroid exposure (4, 5). A study of a US military population found that UC patients were more likely to develop diabetic kidney disease (DKD) compared to Crohn’s disease patients (6). However, it remains uncertain whether DKD is an underlying complication. UC patients are susceptible to drug-associated nephrotoxicity and elevated blood glucose due to medications used in treatment, such as nonsteroidal anti-inflammatory drugs, methotrexate, and 5-aminosalicylic acid (7, 8). Distinguishing between drug-induced diabetic kidney injury and kidney injury due to UC pathogenesis is challenging.

Currently, there are no randomized controlled trials on the relationship between UC and the risk of DKD. Observational studies, which have limitations such as reverse causality, small sample sizes, and confounding factors, leave the relationship uncertain. Mendelian randomization (MR) analyses, similar to randomized controlled trials, use single nucleotide polymorphisms (SNPs) as IVs to infer causal relationships between exposure and outcome (9). Since alleles segregate randomly during meiosis, are not influenced by external factors, and genetic variation occurs before disease onset, the MR method can reduce bias from confounding factors and avoid reverse causation (10). In this study, we used a large-scale genome-wide association study (GWAS) database to analyze the genetic causality between UC and DKD through a bidirectional two-sample MR study. We compared genome-wide genes reported for each trait, extracted the shared genes, and constructed a UC-driven molecular pathway map. This provides a theoretical basis for the clinical treatment and prevention of UC and DKD.

## 2 Methods

### 2.1 Study design

**Figure 1:**
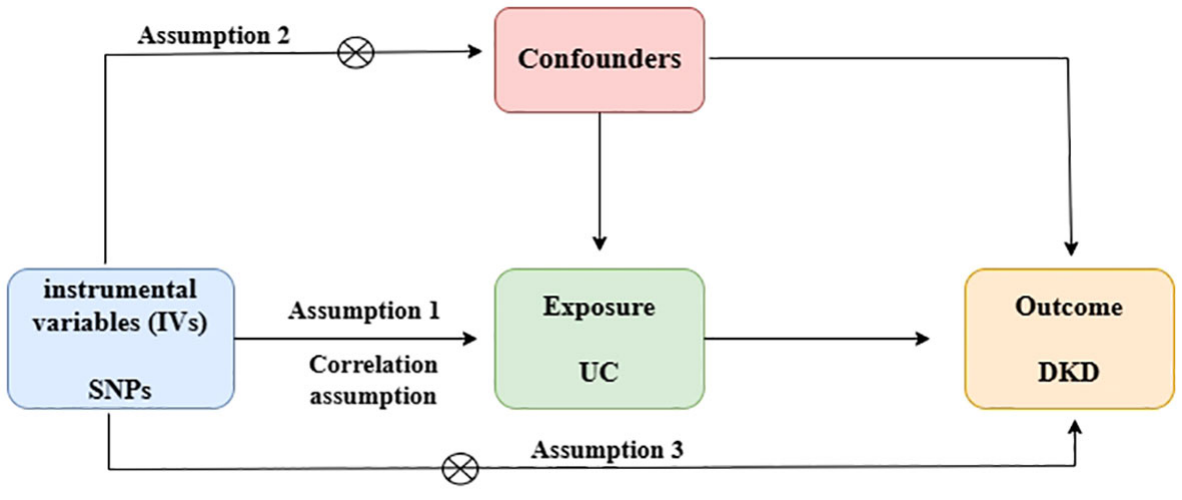
Directed acyclic plot showing MR. Hypothesis 1: Variants are associated with exposure. Hypothesis 2: Variants are not associated with confounders that may bias the exposure-outcome association. Hypothesis 3: Variants affect outcomes only through their association with exposure.

For genetic variation to be a valid tool for causal inference in MR studies, it must satisfy three core assumptions: (1) it must be genuinely associated with the exposure (UC or DKD); (2) it must be independent of exposure-endpoint confounders; and (3) it must affect endpoints only through the exposure, not by any other pathway (9, 11). This MR study utilized a previously published, publicly available, large-scale pooled dataset. All participants provided written informed consent in the original GWAS (**Figure 1**).

### 2.2 Data sources

The data on exposures and outcomes were obtained from BioBank Japan, accessible through the GWAS catalogue (https://www.ebi.ac.uk/gwas). The GWAS data for UC, originating from the East Asian population, was downloaded from this database with ID GCST90018713. It includes 178,689 East Asian individuals (314 cases, 178,375 controls) and covers over 13 million SNPs. Additionally, the GWAS data for DKD, with ID GCST90018612, includes 132,984 East Asian individuals (220 cases, 132,764 controls) and over 13 million SNPs.

### 2.3 Selection of instrumental variables

Selected IVs must satisfy the three basic assumptions of MR analysis outlined in the previous section. Initially, to capture more SNPs significantly associated with exposure, we set the p-value threshold at 5 × 10^-6 for genome-wide significance. Additionally, to mitigate bias from linkage disequilibrium (LD) in the final analysis, we required SNPs significantly associated with exposure to have an r^2 < 0.001 and KB > 10,000. Palindromic SNPs with moderate allele frequencies were excluded from our MR analysis. Furthermore, we assessed the strength of the genetic instrument using the F-statistic for all SNPs, calculated as (β^2/se^2), with an F-statistic for IV in follow-up analysis exceeding 10 (12, 13).

### 2.4 MR analyses

Statistical analyses were conducted using R software (version 4.1.2). MR analyses were conducted using the “TwoSampleMR” package (version 0.5.6). The “MRPRESSO” package (version 1.0) was utilized for MRPRESSO analyses to detect outliers and polytropy. The “MRPRESSO” software package (version 1.0) was employed for MRPRESSO analyses to detect outliers and assess multiple effects.

For causal analyses between exposure and outcome, random-effects inverse variance weighting (IVW) was employed as the primary analytical method. Supplementary methods included MR-Egger, weighted median, simple, and weighted models. Since the indicators used as endpoints were dichotomous variables, we converted ratio estimates to obtain corresponding odds ratios (ORs) and 95% confidence intervals (95% CIs).

### 2.5 Sensitivity analysis

Additionally, we conduct sensitivity analyses, assessing heterogeneity and pleiotropy. Heterogeneity will be assessed using IVW and MR-Egger regression, and the Cochran Q statistic will quantify it. If heterogeneity is present, we will address it by analyzing it using IVW with random effects. Horizontal multivariate validity is crucial for our study because its impact may cause instability in effect estimates. Tests for horizontal pleiotropy involve MR-Egger intercept and MR-PRESSO analysis. MR-Egger intercept estimates the likelihood of horizontal pleiotropy by calculating the intercept term derived from linear regression analyses. MR-PRESSO analyses assess the overall pleiotropy of the study and identify abnormal SNPs that may exhibit horizontal pleiotropy. We configured the software package to conduct MR-PRESSO analysis with 5,000 distributions. The presence of pleiotropy in the study was observed using the Global test. Additionally, we evaluated the robustness of the MR analysis results by comparing effects before and after eliminating aberrant SNPs (12).

### 2.6 Data visualisation

The leave-one-out method was employed to assess the impact of individual SNPs on the results. Publication bias was evaluated by visually examining funnel plots for symmetry, which also served to assess potential directional pleiotropy. Forest plots were utilized to assess effect estimates of genetic variants on UC or DKD. Combined effects were calculated using MR-Egger regression with IVW.

### 2.7 Construction of UC-driven DKD regulatory pathway

To investigate the molecular-level connection between UC and DKD, we retrieved all gene eQTL data from the ieu database (https://gwas.mrcieu.ac.uk/). We filtered the data with a p-value threshold of less than 5e-08 to identify SNPs strongly associated with the exposure factors. Data were filtered based on kb = 10000 and r2 = 0.001. An F-test was applied, and variables with F-test values greater than 10 were removed to eliminate the influence of weak IVs. Next, risk genes associated with UC or DKD were identified through MR analysis. The OR directions obtained from MR Egger, Weighted median, Inverse variance weighted, Simple mode, and Weighted mode methods were consistent, with p-values < 0.05 for Inverse variance weighted. Genes with OR > 1 were classified as high-risk genes, and those with OR < 1 were classified as low-risk genes for the disease. Risk genes related to the intersection of UC and DKD were determined, and forest plots were generated using the forestploter package in R software (version 4.1.2).

### 2.8 GO function and KEGG pathway enrichment analysis of risk genes

We conducted GO function annotation and KEGG pathway enrichment analysis of risk genes associated with UC and DKD using ClusterProfiler (version: 4.0). Gene annotation and signaling pathway catalogs were chosen for GO function annotation and KEGG pathway enrichment analysis based on criteria of p-value < 0.05 and q-value < 0.05.

### 2.9 Co-expression network construction

GeneMANIA (https://genemania.org) is an online analysis tool utilized in this study to construct co-expression and pathway networks of key genes in UC and DKD disease networks. It was employed to identify key targets for treating UC and DKD and predict potential functions of the risk genes.

## 3 Results

### 3.1 MR analysis (causal effect of UC on DKD)

**Figure 2:**
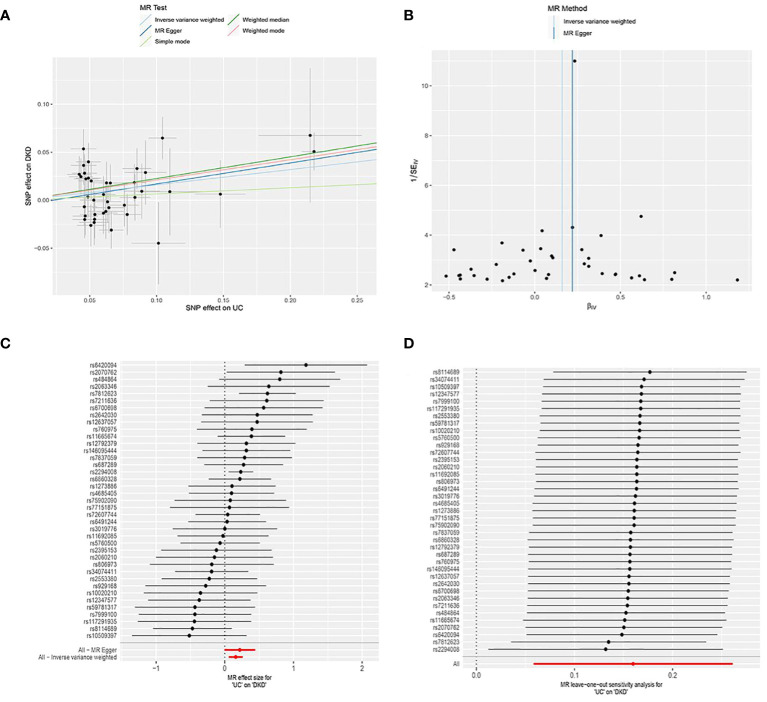
MR analysis of the causal association between genetically predicted ulcerative colitis (UC) and diabetic kidney disease (DKD). **(A)** Scatter plot depicting the causal relationship between genetically predicted UC and DKD. The slope of the line indicates the magnitude of the causal effect. Black dots represent the genetic instruments used in the primary MR analysis. **(B)** Funnel plots illustrating genetically predicted UC’s impact on DKD. **(C)** Forest plot displaying the causal effect of each SNP on DKD risk. **(D)** Leave-one-out plots demonstrating the causal relationship between genetically predicted UC and DKD. MR, Mendelian randomization; SNP, single nucleotide polymorphism.

UC was utilized as the exposure factor and DKD as the outcome variable. After screening, 39 SNPs were identified as IVs (**Supplementary Table 1**), The MR-Egger regression intercept was near 0 (Egger’s intercept = -0.004, p = 0.563), suggesting absence of horizontal pleiotropy in the IVs. Thus, they had minimal impact on the MR analysis results (**Figure 1A**). Due to the absence of horizontal pleiotropy in the IVs, MR analysis using IVW as the primary method revealed a causal relationship between UC and reduced DKD risk (OR = 1.173, 95% CI: 1.059-1.299, p = 0.002), depicted in **Figures 2A, C** and **Table 1**.

**Table 1:** Results from a two-sample Mendelian randomization analysis investigating the causal relationship between ulcerative colitis and the risk of diabetic kidney disease.

Method	SNP(n)	β	SE	OR (95%CI)	p-value
MR Egger	39	0.22	0.11	1.24(0.99, 1.56)	0.06
Weighted median	39	0.23	0.08	1.25(1.08, 1.46)	0.00
Inverse variance weighted	39	0.16	0.05	1.17(1.06, 1.30)	0.00
Simple mode	39	0.06	0.16	1.07(0.78, 1.47)	0.69
Weighted mode	39	0.21	0.09	1.23(1.04, 1.46)	0.02

MR, Mendelian randomisation; SNP, single nucleotide polymorphism; β: allele effect value; SE: standard error.

Heterogeneity among IVs was assessed using IVW and MR-Egger regression. MR-Egger regression yielded Cochran’s Q = 44.332, Q_df = 37, p = 0.189; IVW yielded Cochran’s Q = 44.739, Q_df = 38, p = 0.209 (**Figure 2B**). These results suggest the absence of heterogeneity among the IVs. The MR-PRESSO test revealed no horizontal multivariate validity of the IVs (global test RSSobs = 47.181, p = 0.219), and no outliers were detected. Sensitivity analyses were performed using the leave-one-out method, in which SNPs were sequentially removed to assess their impact on causal associations. This helped determine if any single IVs drove the observed causal effects. The sensitivity analyses demonstrated the robustness of the MR analyses (**Figure 2D**).

### 3.2 Reverse MR analysis (causal effect of DKD on UC)

**Figure 3:**
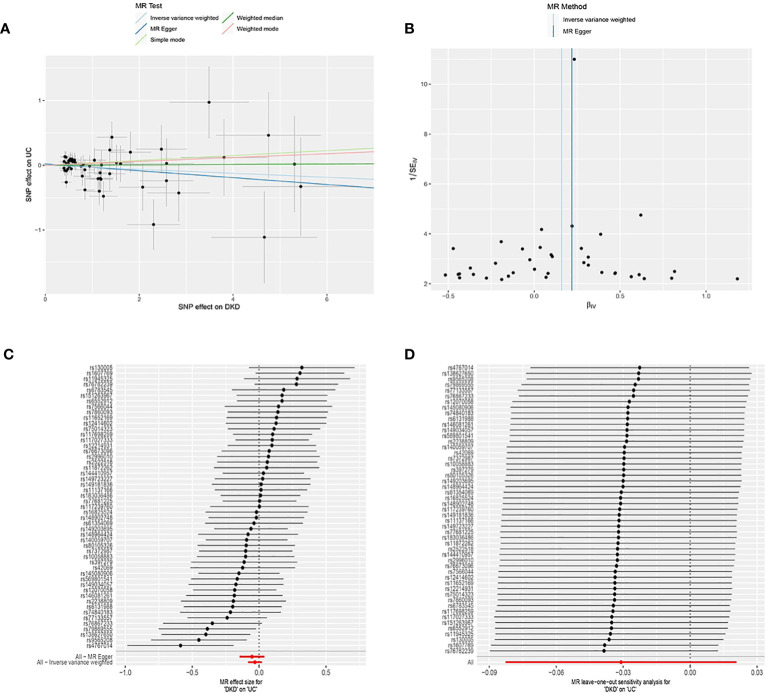
Mendelian Randomization (MR) Analysis of the Causal Relationship between Genetically Predicted Diabetic Kidney Disease (DKD) and Ulcerative Colitis (UC). **(A)** Scatter plot illustrating the causal relationship between genetically predicted DKD and UC. The slope of the line indicates the strength of the causal relationship. Black dots denote genetic instruments used in the primary MR analysis. **(B)** Funnel plots depicting the effect of genetically predicted DKD on UC. **(C) (C)** Forest plot illustrating the causal effect of each SNP on the risk of UC. **(D) (D)** Leave-one-out plots illustrating the causal relationship between genetically predicted DKD and UC.MR, Mendelian randomisation; SNP, single nucleotide polymorphism.

DKD was considered as the exposure, while UC was treated as the outcome. Following screening, 51 SNPs were identified as IVs (**Supplementary Table 2**), all with F values exceeding 10. The horizontal multivariate test indicated no horizontal pleiotropy among the IVs (Egger’s intercept = 0.020, p = 0.574), as depicted in **Figure 3A**. MR analysis results indicated no causal relationship between DKD and an elevated risk of UC (IVW: OR = 0.969, 95% CI: 0.920 to 1.021, p = 0.241), as illustrated in **Table 2** and **Figures 3A, C**. In the heterogeneity test (**Figure 3B**), MR-Egger regression yielded Cochran’s Q = 56.651, Q_df = 49, p = 0.211, and IVW yielded Cochran’s Q = 57.021, Q_df = 50, p = 0.230, suggesting absence of heterogeneity among the IVs. The MR-PRESSO test revealed no horizontal pleiotropy among the IVs (global test RSSobs = 59.250, p = 0.242), and no outliers were identified. Sensitivity analyses confirmed the reliability of the MR analyses (**Figure 3D**).

**Table 2:** Results of Two-Sample Mendelian Randomization Analysis on the Causal Association between Ulcerative Colitis and the Risk of Kidney Disease.

Method	SNP(n)	β	SE	OR (95%CI)	p-value
MR Egger	51	-0.05	0.05	0.95(0.86, 1.04)	0.27
Weighted median	51	0.00	0.04	1.00(0.94, 1.08)	0.93
Inverse variance weighted	51	-0.03	0.03	0.97(0.92, 1.02)	0.24
Simple mode	51	0.04	0.07	1.04(0.90, 1.20)	0.61
Weighted mode	51	0.03	0.07	1.03(0.89, 1.19)	0.69

MR, Mendelian randomisation; SNP, single nucleotide polymorphism; β: allele effect value; SE: standard error.

### 3.3 MR analysis to identify risk genes for UC and DKD

**Figure 4:**
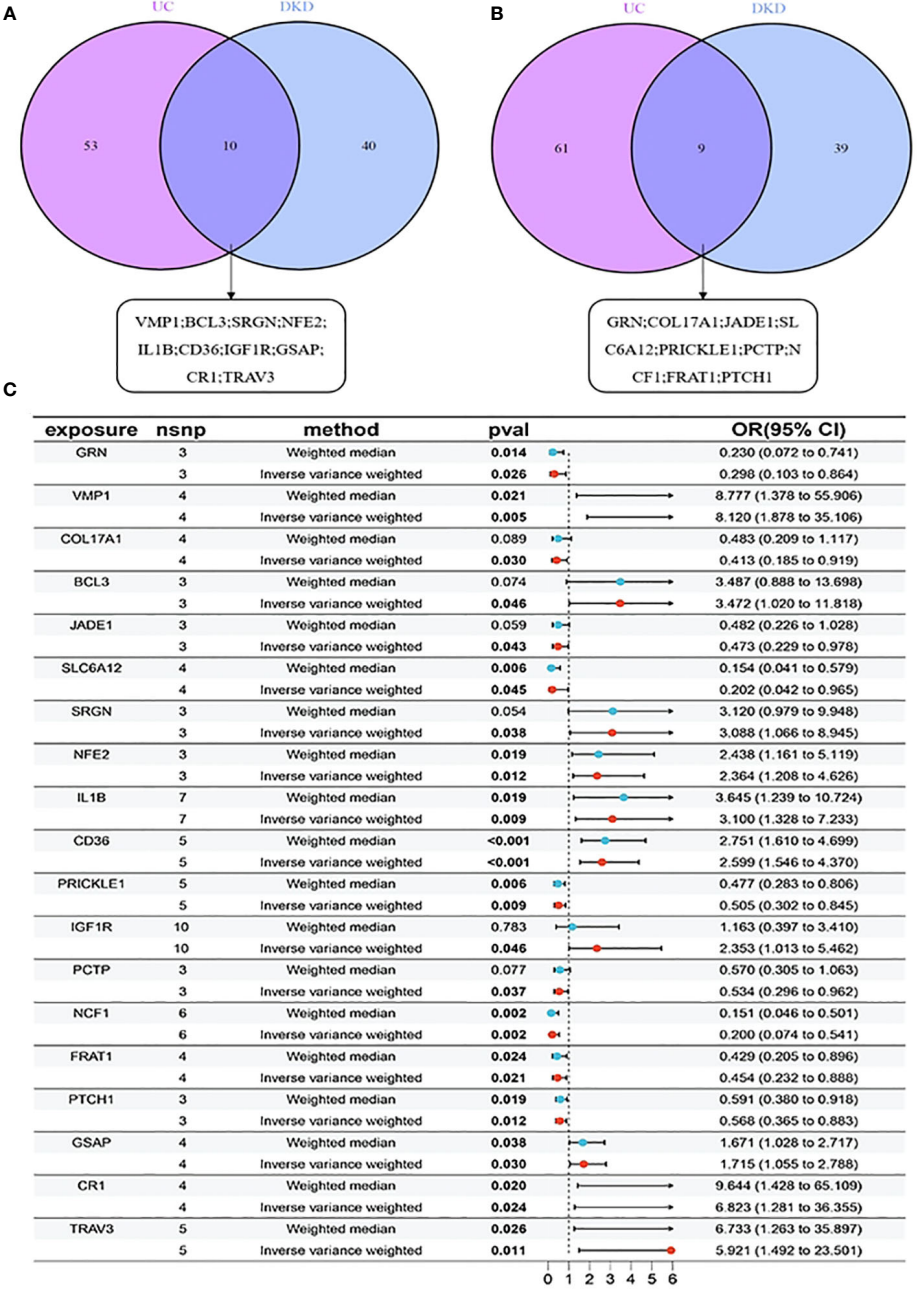
MR analysis for identifying risk genes associated with UC and DKD: **(A)** 10 high-risk genes comparison between UC and DKD; **(B)** 9 low-risk genes comparison between UC and DKD. **(C)** Forest plots illustrating the causal relationship of each SNP with UC risk. mr, Mendelian randomisation; nsnp, number of single nucleotide polymorphisms, pval, p-value.

By analyzing eQTL data for all genes and conducting MR analysis separately for UC and DKD, we identified 133 potential causally linked genes for UC (**Supplementary Table 3**) and 98 potential causally linked genes for DKD (**Supplementary Table 4**). The sets of potential causally linked genes for both diseases were compared, revealing 10 high-risk genes (VMP1; BCL3; SRGN; NFE2; IL1B; CD36; IGF1R; GSAP; CR1; TRAV3) and nine low-risk genes (GRN; COL17A1; JADE1; SLC6A12; PRICKLE1; PCTP; NCF1; FRAT1; PTCH1), as illustrated in **Figures 4A, B**. The forest plot demonstrated a significant association of these 19 risk genes with DKD (p < 0.05 by IVW analysis) (**Figure 4C**).

### 3.4 Enrichment and co-expression analysis of risk genes in UC and DKD

**Figure 5:**
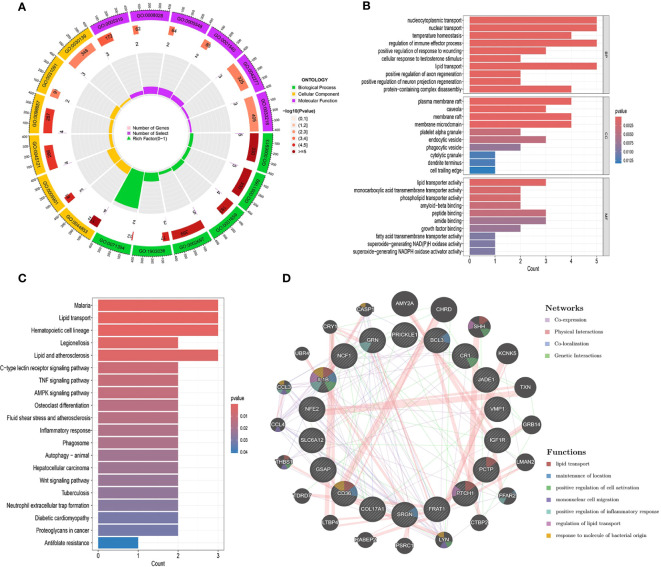
Interaction analysis of UC with DKD risk genes. **(A-C)** Enrichment analysis of GO and KEGG pathways associated with UC and DKD risk genes. **(D)** Gene-gene interaction networks of these risk genes were analyzed using the GeneMANIA database. Risk genes are shown as nodes, and node colors represent gene functions.

All 19 gene targets in the UC and DKD risk gene sets were included and subjected to GO functional enrichment analysis. Based on the GO enrichment analysis of biological processes (BP), cellular composition (CC), and molecular function (MF), the top 10 biological processes with the smallest p-values in each category were selected. These processes include those related to nucleocytoplasmic transport, nuclear transport, temperature regulation, and homeostasis (**Figures 5A, B**). KEGG functional enrichment analyses showed that UC affects DKD mainly through lipid transport, inflammatory response, TNF signaling pathway, AMPK signaling pathway, autophagy, and Wnt signaling pathway. We visualized the relationship between targets and pathways using KEGG pathway enrichment network maps (**Figure 5C**). This suggests that UC may play a role in the treatment of DKD through these pathways.

Co-expression networks of UC and DKD risk genes were obtained using GeneMANIA with an automated weighting method (**Figure 5D**). In the co-expression network, co-expression interactions accounted for 71.82%, physical interactions for 16.25%, co-localization for 6.99%, and gene interactions for 4.93%. The potential functions and possible downstream pathways of the predicted genes were also identified. According to the prediction results, the peripheral genes of risk genes may be associated with lipid transport, positive regulation of inflammatory response, and regulation of lipid transport. These findings are consistent with the results of KEGG analysis.

## 4 Discussion

To the best of our knowledge, this study is the first to explore the causal relationship between UC and DKD using summary statistics from the GWAS Catalog. Our two-way, two-sample MR study showed that genetic susceptibility to UC was associated with a 17.3% increased risk of DKD (Risk ratio, 1.173), whereas genetic susceptibility to DKD was not associated with UC. Additionally, we used various methods to validate the reliability and validity of our results.

The pathogenesis of DKD is related to insulin resistance, pancreatic β-cell dysfunction, decreased glucose clearance, bi-hormonal abnormalities, endoplasmic reticulum stress, intestinal flora dysregulation, and chronic inflammation (14). The pathogenesis of UC, a refractory enteropathy, is related to abnormal immune response, dysregulation of intestinal flora, and mucosal barrier disorders (15). Abnormalities and decreased diversity of microbial metabolites in the human body promote the emergence of DKD-initiating factors such as obesity, diabetes mellitus, hypertension, lipid metabolism disorders, and inflammation. Epidemiological evidence suggests that diabetes mellitus is a common comorbidity of UC, and its prevalence is associated with the severity of UC. Additionally, metformin and acarbose, commonly used to treat T2DM, can also be used to treat UC (16). Diabetes, like other autoimmune diseases, is significantly associated with UC in both pediatric and adult patients. A large case-control study of over 1,200 children with inflammatory bowel disease (IBD), including 488 with UC, showed that UC had a higher prevalence of diabetes compared to controls, with an overall prevalence of 2,049 per 100,000 children (17). UC has the strongest association with diabetes mellitus among autoimmune diseases. The Swedish multigenerational registry enabled the estimation of the association between diabetes and 33 autoimmune and related diseases in parents, offspring, siblings, and twins in a large study. This study showed that the prevalence of diabetes in offspring was associated with 13 autoimmune diseases, including UC (standardized prevalence 1.23), in parents (18). More importantly, data from the Manitoba Health Management Database showed an increased age-adjusted prevalence of various forms of glomerulonephritis in patients with UC (19). Our findings suggest that UC is associated with an increased risk of DKD. This work provides further evidence that UC contributes to DKD, potentially due to alterations in gut flora homeostasis. Studies have shown that compared to healthy individuals, UC patients have decreased levels of beneficial flora such as Lactobacillus and Bifidobacterium, and increased levels of harmful flora such as yeasts and enterococci. This imbalance disrupts biological barriers and induces intense intestinal inflammation, leading to immune function abnormalities (20). The main manifestation is an increased type 2 helper T cell (Th2) immune response and a suppressed type 1 helper T cell (Th1) immune response (21, 22). Intestinal flora is closely associated with extraintestinal non-communicable diseases through intestinal immunity (23, 24). The interaction between gut microorganisms and the kidneys, known as the gut-kidney axis, ensures the balance between intestinal commensals and pathogenic bacteria, maintaining the integrity of the intestinal barrier and normal renal function (25, 26). Healthy kidneys communicate with the gut microbiota through cellular and molecular signals to ensure normal gut microbiota homeostasis (27). In contrast, disruption of this balance due to gut dysbiosis leads to loss of gut barrier integrity, immune cell activation, and cytokine secretion, further deteriorating renal function and symbiosis (28).

Nineteen risk genes for UC and DKD co-morbidity were identified by MR analysis and used to detect GO terms. For GO biological process terms, nucleocytoplasmic transport, nuclear transport, and temperature homeostasis were the most significant. Enhanced nuclear transport may occur in glomerular and tubular cells in diabetic patients and be involved in increased gene expression and nephrosclerosis (29). For cellular components, lipid transporter activity and phospholipid transporter activity ranked high. Individuals with type 2 diabetes mellitus (T2DM) are prone to hyperlipidemia and often experience severe lipid metabolic disturbances (30). In diabetic kidney disease, hyperlipidemia can cause a progressive decline in renal function, marked by albuminuria and reduced glomerular filtration rate (GFR). This occurs through the activation of various intracellular and biochemical pathways, leading to glomerulosclerosis, endothelial dysfunction, oxidative stress, membrane dilation, loss of podocytes, and tubulointerstitial damage (31). Additionally, patients with UC typically have high levels of low-density lipoprotein cholesterol (LDL-C) and triglycerides (TG), and low levels of high-density lipoprotein cholesterol (HDL-C) (32, 33). Koutroumpakis et al. found that persistent dyslipidemia correlates with disease activity in patients with UC (33).

KEGG pathway enrichment analyses were conducted to identify common pathways between DKD and UC. The top 10 KEGG human pathways comprised malaria, lipid transport, haematopoietic cell lineage, legionellosis, lipids and atherosclerosis, C-type lectin receptor signalling pathway, TNF signalling pathway, AMPK signalling pathway, osteoblast differentiation, and fluid shear stress and atherosclerosis. TNF, a cytokine produced by macrophages in response to immunogenic stimuli, belongs to the category of chemotactic cytokines. Changes in its level are closely associated with inflammatory responses. The TNF gene is polymorphic, with base changes mainly occurring at loci TNF-308 and TNF-857, which are associated with the development of UC or DKD (34–36).TNF-308 mutation can enhance TNF activity, leading to increased secretion and imbalance between anti-inflammatory and inflammatory factors in the organism. This results in excessive inflammatory responses and contributes to the development of UC and DKD. Additionally, the TNF-857 mutation is closely related to TNF production, resulting in excessive inflammatory responses in the body and a close association with the occurrence of UC (37). Another study indicated that TNF-857 is correlated with other inflammatory diseases such as DKD, making it an important locus for studying the gene pathogenesis of inflammatory diseases (38–40). TNFα is an inflammatory factor associated with renal disease progression and is known to have two distinct receptors, TNFR1 and TNFR2, with extracellular structural domains capable of being cleaved to form soluble TNFR (sTNFR), which circulates in the bloodstream. The discovery of sTNFR is a significant advancement in DKD progression markers.

In 2012, Niewczas et al. (41) demonstrated that sTNFR could predict renal function decline in patients with type 1 diabetes mellitus (T1DM) and type 2 diabetes mellitus (T2DM) at various disease stages for the first time. After 12 years of follow-up in T1DM patients with normal renal function and urinary albumin levels, it was found that the cumulative incidence of CKD stage 3 in patients in the highest quartile of sTNFR2 was 60%, while in other patients, the incidence ranged from 5% to 19% (42).Furthermore, after adjusting for differences in estimated glomerular filtration rate (eGFR) and albuminuria, several studies have identified elevated concentrations of sTNFR 1 and/or sTNFR 2 as risk factors for DKD progression. These studies were able to optimize the efficacy of clinical prediction models for DKD (43, 44). TNFR may be the most promising biomarker for DKD progression, in addition to eGFR and albuminuria. Moreover, the latest guidelines for pharmacological treatment of UC (45) recommend the use of anti-tumour necrosis factor drugs (infliximab, adalimumab, and golimumab) to induce remission in patients with moderate-to-severe UC who have not adequately responded to, or are intolerant of, conventional treatments. These drugs may also be used to maintain remission in UC patients who have responded to previous drug-induced therapy.

In summary, the mechanisms through which UC contributes to the development of DKD may be multifaceted. However, while these mechanisms are biologically plausible, further well-designed studies are required to explore and confirm the causal relationship between UC and DKD.

Additionally, this study found no causal association between DKD and the risk of UC (IVW: OR=0.969, 95% CI: 0.920-1.021, p=0.241). The complexity of the impact of DKD on gut function may explain this finding. On one hand, elevated toxin levels may exacerbate risk factors for UC, such as inflammatory responses in the body (46). Conversely, patients with DKD promote the secretion of short-chain fatty acids (SCFA) due to gastrointestinal hypodynamia, reduced gastric acid secretion, and bacterial overgrowth in the small intestine (47, 48). SCFA, acting as an anti-inflammatory mediator, alleviate local and systemic inflammation, modify the intestinal microenvironment, and ameliorate UC (49). This bidirectional effect diminishes the impact of DKD on intestinal function and consequently reduces the risk of UC development. Given the limited number of relevant studies, further research is warranted to explore and confirm the relationship between DKD and UC in the future.

The findings of this study carry significant clinical implications. Screening for bowel function, particularly among individuals at high risk of diabetic kidney disease, can aid in identifying specific susceptible individuals for early intervention to mitigate the risk of DKD. Additionally, this study exhibits several strengths. While randomised controlled trials are acknowledged as a research method with high clinical evidence, they are expensive, challenging to follow up, and challenging to execute in practical studies. The two-sample Mendelian randomisation (MR) study design employed in this research can mitigate the influence of confounding factors and reverse causality, thereby mimicking the effects of a randomized controlled trial. Moreover, SNPs highly correlated with exposure (F>10) were chosen as IVs in this study, and multiple sensitivity analyses revealed no evidence of heterogeneity, pleiotropy, or outliers, thus enhancing the robustness of the study findings.

However, this study has several limitations: (i) Despite the use of MR design and rigorous exclusion of known confounders, the results might still be influenced by unaccounted potential confounders. (ii) Since the prevalence of different stroke subtypes varies among ethnicities, only GWAS data from East Asian populations were utilized to prevent population stratification effects, thus limiting the generalizability of the findings to other ethnic groups. (iii) Due to statistical power limitations, a definitive causal association between DKD and UC cannot be entirely ruled out, necessitating future GWAS studies with larger sample sizes for a more conclusive analysis. Additionally, (iv) while some potential biological mechanisms underlying the association between DKD and UC were proposed, further laboratory investigations and exploration of molecular pathways are warranted to elucidate the specific biological connections between the two conditions.

## 5 Conclusion

MR analyses in both cohorts support a causal link between genetic predisposition to ulcerative colitis and the risk of developing diabetic kidney disease, while refuting any influence of diabetic kidney disease on the risk of ulcerative colitis.

## Data Availability

The original contributions presented in the study are included in the article/**Supplementary Material**. Further inquiries can be directed to the corresponding author.
