# Supplementary material for: Elucidating the genetic relationship between ulcerative colitis and diabetic kidney disease: a bidirectional Mendelian randomization study
Source: Front Endocrinol (Lausanne). 2024 Aug 15;15:1435812. doi: 10.3389/fendo.2024.1435812 (PMC11358062; doi:10.3389/fendo.2024.1435812)
Supplement: Supplementary file 5 [file Table1.docx]

**STROBE-MR checklist of recommended items to address in reports of Mendelian randomization studies**^1^ ^2^

| **Item No.** | **Section** | **Checklist item** | **Page No.** | **Relevant text from manuscript** |
| --- | --- | --- | --- | --- |
| 1 | **TITLE and ABSTRACT** | Indicate Mendelian randomization (MR) as the study’s design in the title and/or the abstract if that is a main purpose of the study | 1 | Elucidating the Genetic Relationship Between Ulcerative Colitis and Diabetic kidney disease: A Bidirectional Mendelian Randomization Study |
|  | **INTRODUCTION** |  |  |  |
| 2 | **Background** | Explain the scientific background and rationale for the reported study. What is the exposure? Is a potential causal relationship between exposure and outcome plausible? Justify why MR is a helpful method to address the study question | 2 | Ulcerative colitis (UC), a major form of inflammatory bowel disease (IBD), is a chronic inflammatory condition of the colonic mucosa affecting the rectum and colon to varying degrees. Its pathogenesis is closely linked to genetic susceptibility, microbiota, and immune dysregulation(1). Renal involvement is now common in patients with IBD, including UC(2). A cohort study including renal biopsies from IBD patients showed that out of 896 IBD patients, 218 (24.3%) had renal involvement, of which 161 (73.85%) were UC patients(3). Several national studies have shown that IBD patients have a higher risk of developing type 2 diabetes than the general population, not solely attributable to corticosteroid exposure(4, 5). A study of a US military population found that UC patients were more likely to develop diabetic kidney disease (DKD) compared to Crohn's disease patients(6). However, it remains uncertain whether DKD is an underlying complication. UC patients are susceptible to drug-associated nephrotoxicity and elevated blood glucose due to medications used in treatment, such as nonsteroidal anti-inflammatory drugs, methotrexate, and 5-aminosalicylic acid(7, 8). Distinguishing between drug-induced diabetic kidney injury and kidney injury due to UC pathogenesis is challenging. |
| 3 | **Objectives** | State specific objectives clearly, including pre-specified causal hypotheses (if any). State that MR is a method that, under specific assumptions, intends to estimate causal effects | 2,3 | We used a large-scale genome-wide association study (GWAS) database to analyze the genetic causality between UC and DKD through a bidirectional two-sample MR study. We compared genome-wide genes reported for each trait, extracted the shared genes, and constructed a UC-driven molecular pathway map. |
|  | **METHODS** |  |  |  |
| 4 | **Study design and data sources** | Present key elements of the study design early in the article. Consider including a table listing sources of data for all phases of the study. For each data source contributing to the analysis, describe the following: |  |  |
|  | a) | Setting: Describe the study design and the underlying population, if possible. Describe the setting, locations, and relevant dates, including periods of recruitment, exposure, follow-up, and data collection, when available. | 3,4 | This MR study utilized a previously published, publicly available, large-scale pooled dataset. The data on exposures and outcomes were obtained from BioBank Japan, accessible through the GWAS catalogue (https://www.ebi.ac.uk/gwas). The GWAS data for UC, originating from the East Asian population, was downloaded from this database with ID GCST90018713. It includes 178,689 East Asian individuals (314 cases, 178,375 controls) and covers over 13 million SNPs. Additionally, the GWAS data for DKD, with ID GCST90018612, includes 132,984 East Asian individuals (220 cases, 132,764 controls) and over 13 million SNPs. |
|  | b) | Participants: Give the eligibility criteria, and the sources and methods of selection of participants. Report the sample size, and whether any power or sample size calculations were carried out prior to the main analysis | 4 | The GWAS data for UC, originating from the East Asian population, was downloaded from this database with ID GCST90018713. It includes 178,689 East Asian individuals (314 cases, 178,375 controls) and covers over 13 million SNPs. Additionally, the GWAS data for DKD, with ID GCST90018612, includes 132,984 East Asian individuals (220 cases, 132,764 controls) and over 13 million SNPs. |
|  | c) | Describe measurement, quality control and selection of genetic variants | 4 | Selected IVs must satisfy the three basic assumptions of MR analysis outlined in the previous section. Initially, to capture more SNPs significantly associated with exposure, we set the p-value threshold at 5 × 10^-6 for genome-wide significance. Additionally, to mitigate bias from linkage disequilibrium (LD) in the final analysis, we required SNPs significantly associated with exposure to have an r^2 < 0.001 and KB > 10,000. Palindromic SNPs with moderate allele frequencies were excluded from our MR analysis. Furthermore, we assessed the strength of the genetic instrument using the F-statistic for all SNPs, calculated as (β^2/se^2), with an F-statistic for IV in follow-up analysis exceeding 10(12, 13). |
|  | d) | For each exposure, outcome, and other relevant variables, describe methods of assessment and diagnostic criteria for diseases |  | - |
|  | e) | Provide details of ethics committee approval and participant informed consent, if relevant | 3 | All participants provided written informed consent in the original GWAS |
| 5 | **Assumptions** | Explicitly state the three core IV assumptions for the main analysis (relevance, independence and exclusion restriction) as well assumptions for any additional or sensitivity analysis | 3 | For genetic variation to be a valid tool for causal inference in MR studies, it must satisfy three core assumptions: (1) it must be genuinely associated with the exposure (UC or DKD); (2) it must be independent of exposure-endpoint confounders; and (3) it must affect endpoints only through the exposure, not by any other pathway (9, 11). This MR study utilized a previously published, publicly available, large-scale pooled dataset. |
| 6 | **Statistical methods: main analysis** | Describe statistical methods and statistics used |  |  |
|  | a) | Describe how quantitative variables were handled in the analyses (i.e., scale, units, model) | - | This research does not involve any transformations of quantitative variables. |
|  | b) | Describe how genetic variants were handled in the analyses and, if applicable, how their weights were selected | 4 | For causal analyses between exposure and outcome, random-effects inverse variance weighting (IVW) was employed as the primary analytical method. Supplementary methods included MR-Egger, weighted median, simple, and weighted models. Since the indicators used as endpoints were dichotomous variables, we converted ratio estimates to obtain corresponding odds ratios (ORs) and 95% confidence intervals (95% CIs). |
|  | c) | Describe the MR estimator (e.g. two-stage least squares, Wald ratio) and related statistics. Detail the included covariates and, in case of two-sample MR, whether the same covariate set was used for adjustment in the two samples | 4 | Statistical analyses were conducted using R software (version 4.1.2). MR analyses were conducted using the "TwoSampleMR" package (version 0.5.6). The "MRPRESSO" package (version 1.0) was utilized for MRPRESSO analyses to detect outliers and polytropy. The "MRPRESSO" software package (version 1.0) was employed for MRPRESSO analyses to detect outliers and assess multiple effects. |
|  | d) | Explain how missing data were addressed | - | In this MR analysis, the issue of missing data was not involved. |
|  | e) | If applicable, indicate how multiple testing was addressed | - | In this MR analysis, multiple exposures or multiple outcomes were not involved, so multiple testing was not performed. |
| 7 | **Assessment of assumptions** | Describe any methods or prior knowledge used to assess the assumptions or justify their validity | 5,6 | We filtered the data with a p-value threshold of less than 5e-08 to identify SNPs strongly associated with the exposure factors. Data were filtered based on kb = 10000 and r2 = 0.001. An F-test was applied, and variables with F-test values greater than 10 were removed to eliminate the influence of weak IVs. Next, risk genes associated with UC or DKD were identified through MR analysis. The OR directions obtained from MR Egger, Weighted median, Inverse variance weighted, Simple mode, and Weighted mode methods were consistent, with p-values < 0.05 for Inverse variance weighted. Genes with OR > 1 were classified as high-risk genes, and those with OR < 1 were classified as low-risk genes for the disease. Risk genes related to the intersection of UC and DKD were determined, and forest plots were generated using the forestploter package in R software (version 4.1.2). |
| 8 | **Sensitivity analyses and additional analyses** | Describe any sensitivity analyses or additional analyses performed (e.g. comparison of effect estimates from different approaches, independent replication, bias analytic techniques, validation of instruments, simulations) | 4,5 | we conduct sensitivity analyses, assessing heterogeneity and pleiotropy. Heterogeneity will be assessed using IVW and MR-Egger regression, and the Cochran Q statistic will quantify it. If heterogeneity is present, we will address it by analyzing it using IVW with random effects. Horizontal multivariate validity is crucial for our study because its impact may cause instability in effect estimates. Tests for horizontal pleiotropy involve MR-Egger intercept and MR-PRESSO analysis. MR-Egger intercept estimates the likelihood of horizontal pleiotropy by calculating the intercept term derived from linear regression analyses. MR-PRESSO analyses assess the overall pleiotropy of the study and identify abnormal SNPs that may exhibit horizontal pleiotropy. We configured the software package to conduct MR-PRESSO analysis with 5,000 distributions. The presence of pleiotropy in the study was observed using the Global test. Additionally, we evaluated the robustness of the MR analysis results by comparing effects before and after eliminating aberrant SNPs(12). |
| 9 | **Software and pre-registration** |  |  |  |
|  | a) | Name statistical software and package(s), including version and settings used | 4 | Statistical analyses were conducted using R software (version 4.1.2). MR analyses were conducted using the "TwoSampleMR" package (version 0.5.6). The "MRPRESSO" package (version 1.0) was utilized for MRPRESSO analyses to detect outliers and polytropy. The "MRPRESSO" software package (version 1.0) was employed for MRPRESSO analyses to detect outliers and assess multiple effects. |
|  | b) | State whether the study protocol and details were pre-registered (as well as when and where) |  | This study was not pre-registered with the study protocol and details. |
|  | **RESULTS** |  |  |  |
| 10 | **Descriptive data** |  |  |  |
|  | a) | Report the numbers of individuals at each stage of included studies and reasons for exclusion. Consider use of a flow diagram | 4 | The GWAS data for UC, originating from the East Asian population, was downloaded from this database with ID GCST90018713. It includes 178,689 East Asian individuals (314 cases, 178,375 controls) and covers over 13 million SNPs. Additionally, the GWAS data for DKD, with ID GCST90018612, includes 132,984 East Asian individuals (220 cases, 132,764 controls) and over 13 million SNPs. |
|  | b) | Report summary statistics for phenotypic exposure(s), outcome(s), and other relevant variables (e.g. means, SDs, proportions) | 6,8 | UC was utilized as the exposure factor and DKD as the outcome variable. After screening, 39 SNPs were identified as IVs (Supplementary Table 1), The MR-Egger regression intercept was near 0 (Egger's intercept = -0.004, p = 0.563), suggesting absence of horizontal pleiotropy in the IVs. Thus, they had minimal impact on the MR analysis results (Figure 1A). Due to the absence of horizontal pleiotropy in the IVs, MR analysis using IVW as the primary method revealed a causal relationship between UC and reduced DKD risk (OR = 1.173, 95% CI: 1.059-1.299, p = 0.002), depicted in Figures 2A and 2C and Table 1.  DKD was considered as the exposure, while UC was treated as the outcome. Following screening, 51 SNPs were identified as IVs (Supplementary Table 2), all with F values exceeding 10. The horizontal multivariate test indicated no horizontal pleiotropy among the IVs (Egger's intercept = 0.020, p = 0.574), as depicted in Figure 3A. MR analysis results indicated no causal relationship between DKD and an elevated risk of UC (IVW: OR = 0.969, 95% CI: 0.920 to 1.021, p = 0.241), as illustrated in Table 2 and Figures 3A, 3C. In the heterogeneity test (Figure 3B), MR-Egger regression yielded Cochran's Q = 56.651, Q_df = 49, p = 0.211, and IVW yielded Cochran's Q = 57.021, Q_df = 50, p = 0.230, suggesting absence of heterogeneity among the IVs. The MR-PRESSO test revealed no horizontal pleiotropy among the IVs (global test RSSobs = 59.250, p = 0.242), and no outliers were identified. Sensitivity analyses confirmed the reliability of the MR analyses (Figure 3D). |
|  | c) | If the data sources include meta-analyses of previous studies, provide the assessments of heterogeneity across these studies | 6 | Heterogeneity among IVs was assessed using IVW and MR-Egger regression. MR-Egger regression yielded Cochran's Q = 44.332, Q_df = 37, p = 0.189; IVW yielded Cochran's Q = 44.739, Q_df = 38, p = 0.209 (Figure 2B). |
|  | d) | For two-sample MR:  i.  Provide justification of the similarity of the genetic variant-exposure associations between the exposure and outcome samples  ii.  Provide information on the number of individuals who overlap between the exposure and outcome studies | 2,10 | i. Ulcerative colitis (UC), a major form of inflammatory bowel disease (IBD), is a chronic inflammatory condition of the colonic mucosa affecting the rectum and colon to varying degrees. Its pathogenesis is closely linked to genetic susceptibility, microbiota, and immune dysregulation(1). Renal involvement is now common in patients with IBD, including UC(2). A cohort study including renal biopsies from IBD patients showed that out of 896 IBD patients, 218 (24.3%) had renal involvement, of which 161 (73.85%) were UC patients(3). Several national studies have shown that IBD patients have a higher risk of developing type 2 diabetes than the general population, not solely attributable to corticosteroid exposure(4, 5). A study of a US military population found that UC patients were more likely to develop diabetic kidney disease (DKD) compared to Crohn's disease patients(6). However, it remains uncertain whether DKD is an underlying complication. UC patients are susceptible to drug-associated nephrotoxicity and elevated blood glucose due to medications used in treatment, such as nonsteroidal anti-inflammatory drugs, methotrexate, and 5-aminosalicylic acid(7, 8). Distinguishing between drug-induced diabetic kidney injury and kidney injury due to UC pathogenesis is challenging.  ii. By analyzing eQTL data for all genes and conducting MR analysis separately for UC and DKD, we identified 133 potential causally linked genes for UC (Supplementary Table 3) and 98 potential causally linked genes for DKD (Supplementary Table 4). The sets of potential causally linked genes for both diseases were compared, revealing 10 high-risk genes (VMP1; BCL3; SRGN; NFE2; IL1B; CD36; IGF1R; GSAP; CR1; TRAV3) and nine low-risk genes (GRN; COL17A1; JADE1; SLC6A12; PRICKLE1; PCTP; NCF1; FRAT1; PTCH1), as illustrated in Figures 4A,B. The forest plot demonstrated a significant association of these 19 risk genes with DKD (p < 0.05 by IVW analysis) (Figure 4C). |
| 11 | **Main results** |  |  |  |
|  | a) | Report the associations between genetic variant and exposure, and between genetic variant and outcome, preferably on an interpretable scale | 6,8 | UC was utilized as the exposure factor and DKD as the outcome variable. After screening, 39 SNPs were identified as IVs (Supplementary Table 1), The MR-Egger regression intercept was near 0 (Egger's intercept = -0.004, p = 0.563).  DKD was considered as the exposure, while UC was treated as the outcome. Following screening, 51 SNPs were identified as IVs (Supplementary Table 2), all with F values exceeding 10. The horizontal multivariate test indicated no horizontal pleiotropy among the IVs (Egger's intercept = 0.020, p = 0.574), as depicted in Figure 3A. |
|  | b) | Report MR estimates of the relationship between exposure and outcome, and the measures of uncertainty from the MR analysis, on an interpretable scale, such as odds ratio or relative risk per SD difference | 7,8 | Due to the absence of horizontal pleiotropy in the IVs, MR analysis using IVW as the primary method revealed a causal relationship between UC and reduced DKD risk (OR = 1.173, 95% CI: 1.059-1.299, p = 0.002), depicted in Figures 2A and 2C and Table 1.  MR analysis results indicated no causal relationship between DKD and an elevated risk of UC (IVW: OR = 0.969, 95% CI: 0.920 to 1.021, p = 0.241), as illustrated in Table 2 and Figures 3A, 3C. In the heterogeneity test (Figure 3B), MR-Egger regression yielded Cochran's Q = 56.651, Q_df = 49, p = 0.211, and IVW yielded Cochran's Q = 57.021, Q_df = 50, p = 0.230. The MR-PRESSO test revealed no horizontal pleiotropy among the IVs (global test RSSobs = 59.250, p = 0.242), and no outliers were identified(Figure 3D). |
|  | c) | If relevant, consider translating estimates of relative risk into absolute risk for a meaningful time period | - | The calculation of absolute risk is detailed in Table 1,2. |
|  | d) | Consider plots to visualize results (e.g. forest plot, scatterplot of associations between genetic variants and outcome versus between genetic variants and exposure) | - | The results are visualized in Figure (2-3). |
| 12 | **Assessment of assumptions** |  |  |  |
|  | a) | Report the assessment of the validity of the assumptions | - | Firstly, we selected the SNPs as instrumental variables, which allowing us to perform Mendelian randomization inferences, and the large F statistics indicate that these analyzes will not be affected by weak instrument bias. Secondly, the selected SNPs were ensured to have no association with any confounding factors that could influence the relationship between exposure and outcome. Lastly, the SNPs were confirmed to only impact the outcome through exposure factors. |
|  | b) | Report any additional statistics (e.g., assessments of heterogeneity across genetic variants, such as *I^2^*, Q statistic or E-value) | - | The Cochran's Q test did not detect any heterogeneity of the SNPs, these causal relationships did not show any directional pleiotropy according to the MR-Egger intercept test. |
| 13 | **Sensitivity analyses and additional analyses** |  |  |  |
|  | a) | Report any sensitivity analyses to assess the robustness of the main results to violations of the assumptions | 8,10 | IVW demonstrated a causal relationship between UC and DKD. That is, UC increases the risk of DKD, but the WM method and MR-Egger method did not further confirm the correlation of the other IVW analyses mentioned above, as detailed in Tables 1 and 2. |
|  | b) | Report results from other sensitivity analyses or additional analyses | - | Leave-one-out sensitivity analysis demonstrated the robustness of the MR results, as detailed in Figture 2D and Figture 3D. |
|  | c) | Report any assessment of direction of causal relationship (e.g., bidirectional MR) | - | We used bidirectional MR analysis, The forward analysis proved the causal relationship between UC and DKD, as detailed in Table 1. However, no reverse causal relationship between DKD and DKD was found. SeeTable 2 for details. |
|  | d) | When relevant, report and compare with estimates from non-MR analyses |  | This study does not involve non-MR studies |
|  | e) | Consider additional plots to visualize results (e.g., leave-one-out analyses) | - | To visualize the MR analysis, forest plots, scatter plots, and leave-one-out plots were generated using the data analysis function of the Rstudio platform, as detailed in Figture 2 and Figture 3. |
|  | **DISCUSSION** |  |  |  |
| 14 | **Key results** | Summarize key results with reference to study objectives | 13,14 | Our study revealed a significant causal association between genetic susceptibility to UC and an increased risk of DKD. Specifically, individuals with a genetic predisposition to UC exhibited a 17.3% higher risk of developing DKD. Conversely, no causal relationship was found between DKD and the risk of UC. |
| 15 | **Limitations** | Discuss limitations of the study, taking into account the validity of the IV assumptions, other sources of potential bias, and imprecision. Discuss both direction and magnitude of any potential bias and any efforts to address them | 18 | (i) Despite the use of MR design and rigorous exclusion of known confounders, the results might still be influenced by unaccounted potential confounders. (ii) Since the prevalence of different stroke subtypes varies among ethnicities, only GWAS data from East Asian populations were utilized to prevent population stratification effects, thus limiting the generalizability of the findings to other ethnic groups. (iii) Due to statistical power limitations, a definitive causal association between DKD and UC cannot be entirely ruled out, necessitating future GWAS studies with larger sample sizes for a more conclusive analysis. Additionally, (iv) while some potential biological mechanisms underlying the association between DKD and UC were proposed, further laboratory investigations and exploration of molecular pathways are warranted to elucidate the specific biological connections between the two conditions. |
| 16 | **Interpretation** |  |  |  |
|  | a) | Meaning: Give a cautious overall interpretation of results in the context of their limitations and in comparison with other studies | 13 | The results demonstrate a potential causal relationship between UC and DKD. |
|  | b) | Mechanism: Discuss underlying biological mechanisms that could drive a potential causal relationship between the investigated exposure and the outcome, and whether the gene-environment equivalence assumption is reasonable. Use causal language carefully, clarifying that IV estimates may provide causal effects only under certain assumptions | 14 | Our findings suggest that UC is associated with an increased risk of DKD. This work provides further evidence that UC contributes to DKD, potentially due to alterations in gut flora homeostasis. Studies have shown that compared to healthy individuals, UC patients have decreased levels of beneficial flora such as Lactobacillus and Bifidobacterium, and increased levels of harmful flora such as yeasts and enterococci. This imbalance disrupts biological barriers and induces intense intestinal inflammation, leading to immune function abnormalities (20). |
|  | c) | Clinical relevance: Discuss whether the results have clinical or public policy relevance, and to what extent they inform effect sizes of possible interventions | 17,18 | The findings of this study carry significant clinical implications. Screening for bowel function, particularly among individuals at high risk of diabetic kidney disease, can aid in identifying specific susceptible individuals for early intervention to mitigate the risk of DKD. Additionally, this study exhibits several strengths. While randomised controlled trials are acknowledged as a research method with high clinical evidence, they are expensive, challenging to follow up, and challenging to execute in practical studies. The two-sample Mendelian randomisation (MR) study design employed in this research can mitigate the influence of confounding factors and reverse causality, thereby mimicking the effects of a randomized controlled trial. Moreover, SNPs highly correlated with exposure (F>10) were chosen as IVs in this study, and multiple sensitivity analyses revealed no evidence of heterogeneity, pleiotropy, or outliers, thus enhancing the robustness of the study findings. |
| 17 | **Generalizability** | Discuss the generalizability of the study results (a) to other populations, (b) across other exposure periods/timings, and (c) across other levels of exposure | 18 | (i) Despite the use of MR design and rigorous exclusion of known confounders, the results might still be influenced by unaccounted potential confounders. (ii) Since the prevalence of different stroke subtypes varies among ethnicities, only GWAS data from East Asian populations were utilized to prevent population stratification effects, thus limiting the generalizability of the findings to other ethnic groups. (iii) Due to statistical power limitations, a definitive causal association between DKD and UC cannot be entirely ruled out, necessitating future GWAS studies with larger sample sizes for a more conclusive analysis. Additionally, (iv) while some potential biological mechanisms underlying the association between DKD and UC were proposed, further laboratory investigations and exploration of molecular pathways are warranted to elucidate the specific biological connections between the two conditions. |
|  | **OTHER INFORMATION** |  |  |  |
| 18 | **Funding** | Describe sources of funding and the role of funders in the present study and, if applicable, sources of funding for the databases and original study or studies on which the present study is based | 18 | This study is supported by the Chongqing Postdoctoral Science Foundation Program (No. CSTB2023NSCQ-BHX0087) and Chongqing Postdoctoral Research Program Special Grant (No. 2022CQBSHTB3001). |
| 19 | **Data and data sharing** | Provide the data used to perform all analyses or report where and how the data can be accessed, and reference these sources in the article. Provide the statistical code needed to reproduce the results in the article, or report whether the code is publicly accessible and if so, where | 18,19 | The original contributions presented in the study are included in the article/Supplementary Material. Further inquiries can be directed to the corresponding author. |
| 20 | **Conflicts of Interest** | All authors should declare all potential conflicts of interest | 20 | The authors declare that the research was conducted in the absence of any commercial or financial relationships that could be construed as a potential conflict of interest. |

This checklist is copyrighted by the Equator Network under the Creative Commons Attribution 3.0 Unported (CC BY 3.0) license.

1. Skrivankova VW, Richmond RC, Woolf BAR, Yarmolinsky J, Davies NM, Swanson SA, et al. Strengthening the Reporting of Observational Studies in Epidemiology using Mendelian Randomization (STROBE-MR) Statement. JAMA. 2021;under review.

2. Skrivankova VW, Richmond RC, Woolf BAR, Davies NM, Swanson SA, VanderWeele TJ, et al. Strengthening the Reporting of Observational Studies in Epidemiology using Mendelian Randomisation (STROBE-MR): Explanation and Elaboration. BMJ. 2021;375:n2233.
